# White Matter Microstructure Underlies the Effects of Sleep Quality and Life Stress on Depression Symptomatology in Older Adults

**DOI:** 10.3389/fnagi.2020.578037

**Published:** 2020-11-13

**Authors:** Changhong Li, Jan Schreiber, Nora Bittner, Shumei Li, Ruiwang Huang, Susanne Moebus, Andreas Bauer, Svenja Caspers, David Elmenhorst

**Affiliations:** ^1^Institute of Neuroscience and Medicine (INM-2), Forschungszentrum Jülich, Jülich, Germany; ^2^Department of Neurophysiology, Institute of Zoology, RWTH Aachen University, Aachen, Germany; ^3^Institute of Neuroscience and Medicine (INM-1), Forschungszentrum Jülich, Jülich, Germany; ^4^Institute for Anatomy I, Medical Faculty, Heinrich Heine University Düsseldorf, Düsseldorf, Germany; ^5^German Center for Neurodegenerative Diseases (DZNE), Bonn, Germany; ^6^Center for Studies of Psychological Application, Guangdong Key Laboratory of Mental Health and Cognitive Science, School of Psychology, South China Normal University, Guangzhou, China; ^7^Institute of Medical Informatics, Biometry and Epidemiology, University of Duisburg-Essen, Essen, Germany; ^8^Department of Neurological, Medical Faculty, Heinrich Heine University Düsseldorf, Düsseldorf, Germany; ^9^JARA-BRAIN, Jülich-Aachen Research Alliance, Jülich, Germany

**Keywords:** sleep quality, diffusion tensor imaging, tract-based spatial statistics, mediation analysis, depression and stress, axonal and myelin degeneration

## Abstract

Sleep complaints are the most prevalent syndromes in older adults, particularly in women. Moreover, they are frequently accompanied with a high level of depression and stress. Although several diffusion tensor imaging (DTI) studies reported associations between sleep quality and brain white matter (WM) microstructure, it is still unclear whether gender impacts the effect of sleep quality on structural alterations, and whether these alterations mediate the effects of sleep quality on emotional regulation. We included 389 older participants (176 females, age = 65.5 ± 5.5 years) from the 1000BRAINS project. Neuropsychological examinations covered the assessments of sleep quality, depressive symptomatology, current stress level, visual working memory, and selective attention ability. Based on the DTI dataset, the diffusion parameter maps, including fractional anisotropy (FA), mean diffusivity (MD), axial diffusivity (AD), and radial diffusivity (RD), were calculated and normalized to a population-specific FA template. According to the global Pittsburgh Sleep Quality Index (PSQI), 119 poor sleepers (PSQI: 10∼17) and 120 good sleepers (PSQI: 3∼6) were identified. We conducted a two by two (good sleepers/poor sleepers) × (males/females) analysis of variance by using tract-based spatial statistics (TBSS) and JHU-ICBM WM atlas-based comparisons. Moreover, we performed a voxel-wise correlation analysis of brain WM microstructure with the neuropsychological tests. Finally, we applied a mediation analysis to explore if the brain WM microstructure mediates the relationship between sleep quality and emotional regulation. No significant differences in brain WM microstructure were detected on the main effect of sleep quality. However, the MD, AD, and RD of pontine crossing tract and bilateral inferior cerebellar peduncle were significant lower in the males than females. Voxel-wise correlation analysis revealed that FA and RD values in the corpus callosum were positively related with depressive symptomatology and negatively related with current stress levels. Additionally, we found a significantly positive association between higher FA values in visual-related WM tracts and better outcomes in a visual pattern recognition test. Furthermore, a mediation analysis suggested that diffusion metrics within the corpus callosum partially mediated the associations between poor sleep quality/high stress and depressive symptomatology.

## Introduction

Poor sleep quality has been associated with changes of brain structure and function by using advanced magnetic resonance imaging (MRI) techniques (Sexton et al., 2014; Spira et al., 2016; Amorim et al., 2018). Of these MRI modalities, diffusion tensor imaging (DTI) (Le Bihan et al., 2001), which quantifies three-dimensional displacements of water molecules along axons, reflects white matter (WM) microstructure connecting distributed brain regions. Several diffusion metrics, including fractional anisotropy (FA), mean diffusivity (MD), axial diffusivity (AD), and radial diffusivity (RD), were developed to evaluate brain tissue characteristics (e.g., fiber density, myelination, and axon diameter). In brief, FA indicates the degree of anisotropic diffusion and MD is an orientation-independent index by averaging three diffusion eigenvalues. AD and RD separately reflects the integrity of axonal and myelin microstructure. Previous DTI studies (Yaffe et al., 2016; Khalsa et al., 2017; Sexton et al., 2017) showed that poor sleep quality was primarily related to impaired WM integrity in tracts connecting frontal, temporal and subcortical regions. These WM changes were accompanied by attention and memory deficits, as well as emotional dysregulations. For instance, in 448 older adults, Sexton et al. (2017) found that poor self-reported sleep quality was significantly correlated with reduced global FA and increased global AD and RD. By using tract-based spatial statistics (TBSS), they further reported disrupted frontal-subcortical tracts, which was consistent with a previous study (Li et al., 2016) in patients with primary insomnia. However, the generalizability of abovementioned results was restricted because of the high fraction of men within the participants.

As known, sleep complaints tend to occur more frequently in women (Bei et al., 2015; Mong and Cusmano, 2016; Hinz et al., 2017), which might be affected by gender-specific variations in brain organization. Actually, large amounts of MRI research (Ingalhalikar et al., 2014; Kanaan et al., 2014; Cox et al., 2016; Ritchie et al., 2018; Lotze et al., 2019) have addressed the gender differences in brain structural and functional architectures, as well as morphometry. For example, based on 5216 participants of UK Biobank datasets, Ritchie et al. (2018) found higher FA values in males than in females in widespread deep WM tracts, such as cerebellar peduncle, genu of corpus callosum, and anterior limb of internal capsule. Lotze et al. (2019) presented a larger local gray matter volume (GMV) of frontal and temporal cortices but smaller GMV of subcortical areas in females compared to male in a total of 2838 adult subjects. Therefore, it is essential to evaluate the interaction effects between gender differences (males/females) and sleep quality (high/poor) on brain WM microstructure. Additionally, sleep quality is associated with emotion processing in a bidirectional fashion so that emotional dysregulations (e. g., stressful and depressive syndromes) could aggravate sleep complaints, and vice versa (Baglioni et al., 2010, 2011; Potvin et al., 2014; Becker et al., 2017). Recently, based on 1017 healthy participants of the Human Connectome Project, Cheng et al. (2018) found that both poor sleep quality and high depression scores were significantly positively correlated with increased functional connectivity that involved prefrontal, hippocampal, and amygdala regions. A mediation analysis revealed that the level of connectivity in these regions partially mediated the association of depressive symptoms with sleep quality. Nevertheless, most previously published DTI studies considered depression scores as covariates without exploring the role of sleep quality.

Therefore, we intended to evaluate how sleep quality and gender differences (males/females) influence whole brain WM microstructure, as well as how these WM tracts mediate the associations between emotional dysregulation and poor sleep quality in older adults. With the application of both TBSS and atlas-based comparisons, we applied two-way repeated analysis of variance (ANOVA) (two factors with sleep quality and gender differences) to compare the significant differences in diffusion metrics of whole brain microstructures. A voxel-wise correlation approach was used to examine relations between brain WM tracts and outcomes of neuropsychological measurements. Lastly, with mediation analysis, we tested the mediation effects among sleep quality, emotional regulation (depression/stress), and brain WM microstructure.

## Materials and Methods

### Participants

All datasets were selected from the 1000BRAINS project that aims to study the consequences of aging on brain structure, cognitive function, and behavioral performance in an older population-based German cohort (Schmermund et al., 2002; Caspers et al., 2014). From an initial sample of 682 participants at the time of initiation of this study, 293 were excluded according to the following criteria: (i) a participant is suffering or has any history of neurological or neuropsychiatric disorders over the past 10 years, (ii) at least three variables of the cognitive and neuropsychological examinations were not acquired, (iii) age is less than 55 or larger than 80 years, and (iv) failed acquisition of MRI data or serious artifacts. As a result, we included 389 healthy older subjects (176 females, mean age: 65.5 ± 5.5 years) for further analysis. In addition, we measured the Body Mass Index (BMI) and used self-report questionnaires and interviews to record the education (the period of received education in school or university prior to commencing the work), physical activity (hours/week) and habitual coffee consumption. Informed consent was obtained from each participant and the ethical application was approved by the local Ethical Committee (University Hospital, Duisburg-Essen, Germany).

### MRI Data Acquisition

All MRI data were collected on a 3T Siemens TRIO Tim MRI scanner with a 32-channel phased-array head coil. For each participant, high resolution brain structural images were acquired using a T1-weighted 3D MP-RAGE (magnetization prepared rapid acquisition gradient echo) with the following parameters: repetition time (TR) = 2,250 s, echo time (TE) = 3.03 ms, inversion time = 900 ms, flip angle = 9°, filed of view (FOV) = 256 mm × 256 mm, data matrix = 256 × 256, 176 sagittal slices, and voxel resolution = 1 mm × 1 mm × 1 mm. The DTI dataset of each participant was acquired using a twice refocused spin-echo diffusion-weighted EPI sequence with the following parameters: TR = 6,300 ms, TE = 81 ms, 60 directions with *b* = 1000 s/mm^2^, 7 b0 volumes, FOV = 216 mm × 216 mm × 132 mm, 55 interleaved transversal slices without inter-slice gap, and voxel resolution = 2.4 mm × 2.4 mm × 2.4 mm.

### MRI Pre-processing

For each participant, the DTI data underwent correction for motion artifacts including interpolation of slices with signal dropouts by eddy in FSLv6.0^1^ (Andersson and Sotiropoulos, 2016; Andersson et al., 2017). Visual quality control (QC) was performed to check for ghosting, remaining signal dropouts or very noisy T1-weighted/DTI data. Tissue probability maps for gray matter, WM and cerebrospinal fluid (CSF) were then estimated from T1-weighted data using CAT12^2^ which ran in SPM12 software^3^. This step also created a brain mask by adding up the three tissue probability maps, thresholding them at 0.5 and filling small holes. This brain mask was then used to extract the brain from the T1-weighted data. Then, the T1-weighted images were corrected for its bias field, rigidly aligned with the MNI152 template space and resampled to 1.25 mm isotropic voxel size. With *fslmaths* command, we also calculated the total intracranial volume (TIV) by sum of the above three tissue probability maps, which was regarded as a nuisance variable during further statistical comparisons.

For the DTI dataset, the first b0 images were extracted and rigidly aligned to the T1-weighted data using mutual information as the cost function. Then, the DTI datasets were transferred to each T1-weighted individual space (resampling to 1.25 mm isotropic voxel size) with corresponding transformations based on the alignment of the corresponding b0 volumes. B-vectors were rotated according to the transformations. The diffusion tensor-derived maps were computed using *dtifit* in FSLv6.0, including FA, MD (MD = (λ1 + λ2 + λ3)/3), AD (AD = λ1), and RD (RD = (λ2 + λ3)/2) maps.

### Tract-Based Statistical Statistics (TBSS) Approach

In this section, we adopted the processing pipeline from Schwarz et al. (2014) to improve our DTI registration through Advanced Normalization Tools (ANTs)^4^. First, the binary erosion step with a 3 × 3 × 3 voxel kernel was conducted to remove “halo” voxels. Then, the FA images of 50 randomly sampled participants were rigidly registered to FMRIB58_FA template and averaged for creating an initial FA template with 1 mm × 1 mm × 1 mm resolution in the MNI152 standard space. Next, a group-averaged FA template was built from all subjects’ FA maps using Buildtemplateparallel.sh with cross correlations and four iterations. With *antsRegistrationSyN.sh*, the diffusion parameter maps of each subject were non-linearly transformed to the group-averaged FA template. After averaging all normalized images, a skeleton of the whole brain WM tracts was extracted at a threshold of FA > 0.2 and then back-projected to the diffusion parameter maps of each individual subject. At last, the diffusion parameter maps (FA, MD, AD, and RD) were merged into a single 4D image for further statistical comparisons. In addition, the mean values of diffusion metrics in all 48 tracts that were derived from JHU-ICBM WM labels atlas^5^ were computed.

### Sleep Quality and Other Neuropsychological Measurements

Prior to the MRI scanning, Pittsburgh Sleep Quality Index (PSQI) and Beck Depression Inventory (BDI) were separately applied to access sleep quality and depression severity (Beck et al., 1988; Buysse et al., 1989). In details, the PSQI is an established self-report questionnaire that evaluates sleep quality over the past 1 month. It consists of 19 items, creating seven components that can be summarized as one global PSQI score. BDI is a 21-items self-report inventory for assessing depressive symptoms. At the beginning and end of the test day, the current stress levels of all participants were measured using a short questionnaire named “Kurzfragebogen zur aktuellen Beanspruchung” (KAB) (“Short Questionnaire on Current Stress Levels”). We classified the participants into poor sleepers (PS) (PSQI > 9; range 10∼17) and good sleepers (GS) (PSQI < 7; range 3∼6), which resulted in 120 GS (43 females, mean age = 65.5 ± 5.6 years old) and 119 PS (62 females, mean age = 65.5 ± 5.7 years old) (Table 1). The cutoff was chosen so that the proportion of poor and good sleepers approximately corresponded to the upper and lower quartile of the entire population, respectively.

**TABLE 1:** Demographical and neuropsychological performance data of all participants (mean ± standard derivation).

	Poor sleepers (PS)		Good sleepers (GS)		*p*-value		
	Male	Female	Male	Female	Sleep quality	Gender	Sleep quality × Gender
Number of participants	57	62	77	43	0.011*	NA	NA
Age (years)	65.88 (6.13)	65.10 (5.24)	65.99 (5.51)	64.72 (5.59)	0.85	0.17	0.74
Education (years)	9.89 (1.93)	9.71 (1.81)	10.01 (2.14)	9.74 (1.85)	0.65	0.30	0.99
Pittsburgh Sleep Quality Index (PSQI)	11.56 (1.44)	12.42 (2.01)	5.09 (0.88)	5.02 (0.80)	NA	0.032*	NA
Beck Depression Inventory (BDI)	7.20 (5.91)	6.77 (3.8)	3.84 (2.51)	5.65 (3.77)	<0.001*	0.20	0.039*
Habitual intake of coffee (cups)	4.76 (0.74)	4.47 (1.03)	4.86 (0.64)	4.36 (1.28)	0.98	0.003*	0.42
Physical activity (hours/week)	3.53 (2.68)	3.13 (2.63)	4.43 (5.73)	5.07 (4.44)	0.005*	0.98	0.24
Body Mass Index (BMI)	26.46 (3.71)	28.61 (5.54)	27.27 (5.76)	25.88 (4.29)	0.84	0.27	0.31
Total Intracranial Volume (mm^3^)	1,556,080 (111,287)	1,375,386 (87,567)	1,390,511 (83,202)	15,658,825 (111,360)	0.35	<0.001*	0.84
Current stress levels (Kurzfragebogen zur aktuellen Beanspruchung, KAB1)	26.75 (6.09)	28.61 (5.88)	23.81 (5.94)	26.47 (5.97)	0.001*	0.005*	0.61
Current stress levels (Kurzfragebogen zur aktuellen Beanspruchung, KAB2)	26.44 (5.63)	28.90 (6.58)	23.30 (5.74)	25.98 (5.71)	<0.001*	0.001*	0.89
Trail Making Test (part A, TMT-A)	39.22 (12.52)	42.26 (16.09)	39.02 (12.38)	38.32 (11.98)	0.24	0.51	0.29
Selective attention task (Alters-Konzentrations-Test, AKT)	34.08 (10.22)	34.56 (8.26)	32.62 (7.79)	35.03 (9.09)	0.67	0.21	0.41
Visual pattern (VPT, Juelich version)	8.18 (1.76)	6.77 (1.43)	8.40 (1.73)	6.88 (1.61)	0.44	<0.001*	0.79
Verbal working memory (Zahlennachsprechen, ZNS)	11 (2.05)	10.63 (1.59)	10.71 (1.70)	10.42 (1.72)	0.29	0.16	0.87
Corsi Block-Tapping-Test (CBT)	10.33 (1.66)	9.85 (1.35)	10.56 (1.72)	10.12 (1.48)	0.24	0.03*	0.93

Abbreviations: NA, not applicable. *Significant level at *p* < 0.05 level.

Participants underwent the following neuropsychological tests: (i) a selective attention task [Alters-Konzentrations-Test (AKT)], in which the participants were asked to find and cross out a target item to access attention ability; (ii) a Trail Making Test (part A, TMT-A), which is defined as time (in sec.) needed to connect randomly arranged digits in ascending order reflecting processing speed. (iii) To evaluate working memory performance, a visual spatial working memory [Corsi Block-Tapping Test (CBT)], (iv) a visual pattern test (Juelich version, VPT), and (v) a verbal working memory test [Zahlennachsprechen (ZNS)] were employed. Briefly, CBT counts the number of tapped blocks in a given sequence which were repeated correctly (sum score forward and backward). VPT indicates the total number of memorized patterns of a matrix of black and white squares with increasing complexity and ZNS represents the number of digits in a sequence which were correctly repeated (sum score forward and backward). For more interpretations, please refer to Jockwitz et al. (2017a).

### Statistical Comparisons and Mediation Analysis

For the demographic information, we first applied χ^2^ test to examine the statistical significance of gender differences on sleep quality. Then, an independent two-sample *t*-test was used for testing the differences of PSQI scores between the males and females. For other variables (Age, Education, Habitual intake of coffee, Physical activity, BMI, TIV, KAB, BDI, VPT, TMT, AKT, CBT, and ZNS), we conducted an ANOVA approach to calculate the main effects and the interaction of sleep quality (GS and PS) and gender differences (males and females). All statistical comparisons were considered significantly different at *p* < 0.05 level.

Using the TBSS approach, we also performed an ANOVA to test the main effects and the interaction of sleep quality (GS and PS) and gender differences (males and females) in the whole brain WM skeleton. During the comparisons, age, education, habitual coffee consumption, physical activity, BMI, and brain TIV were taken as covariances. Then, the diffusion metrics of 239 participants were examined by voxel-wise correlation analysis between brain WM skeleton and neuropsychological examinations. Notably, gender was additionally regressed out during this step. All above mentioned statistical analysis were conducted by *randomize* (5,000 permutations) with a Threshold-Free Cluster Enhancement (TFCE) approach. Moreover, the significant inferences were corrected using family wise error rates (FWER) at *p* < 0.05 level. For the JHU-ICBM atlas-based comparisons, we replicated these procedures and examined their correlations with outcomes of neuropsychological tests (Bonferroni correction at *p* < 0.001) in SPSS22^6^.

Following the voxel-wise correlation analysis, we selected those voxels which were significantly correlated with psychological questionnaires (BDI scores, KAB scores, or PSQI scores), and extracted the mean values of diffusion metrics within corresponding clusters. Then, a standard three-variable mediation analysis proposed by MacKinnon et al. (2007) was used to identify potential mediation effects of diffusion metrics in brain WM tracts among depression, stress, and sleep quality. In brief, we calculated the path coefficients between the independent (X), dependent (Y), and mediator (M) variables in terms of three regression equations. At the beginning, we determined whether path coefficients a (effect of X on M), b (effect of M on Y), and c (effect of X on Y) were all significant. If these criteria were met, we tested the significance of the indirect (i.e., mediated) effect, indicated by the regression coefficients a and b (bootstrapping 5,000 times). Finally, we computed the direct effect (c’), the indirect effect (ab), and the total effect (c = c’ + ab) with AMOS^7^.

## Results

### Demographical Information

Table 1 shows the demographical information in our sample of 239 participants and the statistical comparisons of poor and good sleepers (according to PSQI) regarding their neuropsychological performance. In details, main effects of both sleep quality and gender were only significantly on the stress level (KAB scores). Then, we observed that the males had significantly lower self-reported global PSQI scores, higher habitual intake of coffee, larger TIV, and better performances of working memory (VPT and CBT scores) than the females. Meanwhile, the good sleepers had significant lower depression level (BDI scores), more physical activity, and higher numbers of the males compared to the poor sleepers. Lastly, the interaction of gender differences and sleep quality was significant for the depression level (BDI scores). Age, education, BMI, selective attention ability (TMT-A and AKT scores), or verbal working memory ability (ZNS scores) did not show any significant differences.

### Statistical Results of Two-Way ANOVA Analysis

Using TBSS analysis, neither main effects nor the interaction of sleep quality and gender differences were statistically significant. Using the JHU-ICBM atlas-based comparisons, we found significantly lower mean MD, mean AD, and mean RD values of the pontine crossing tract and bilateral inferior cerebellar peduncle in the males in comparison to females (Table 2, Bonferroni correction at *p* < 0.001).

**TABLE 2:** Gender differences on the JHU-ICBM white matter atlas.

	Mean diffusivity (10^–4^ mm^2^/sec)			Axial diffusivity (10^–4^ mm^2^/sec)			Radial diffusivity (10^–4^ mm^2^/sec)		
	Male	Female	*p*-value	Male	Female	*p*-value	Male	Female	*p*-value
Pontine crossing tract	6.55 ± 0.05	6.88 ± 0.06	2.7e-04*	9.53 ± 0.06	10.04 ± 0.08	1.2e-05*	5.06 ± 0.05	5.30 ± 0.06	0.0067
Right inferior cerebellar peduncle	7.26 ± 0.05	7.68 ± 0.06	2.0e-06*	11.05 ± 0.06	11.61 ± 0.07	1.0e-06*	5.36 ± 0.05	5.72 ± 0.06	2.7e-05*
Left inferior cerebellar peduncle	7.31 ± 0.06	7.66 ± 0.07	8.5e-04*	11.14 ± 0.07	11.51 ± 0.08	0.0014	5.38 ± 0.05	5.67 ± 0.06	0.0023

*Significance level at *p* < 0.001.

### Associations of White Matter With Depressive and Stress-Related Symptomatology

**FIGURE 1:**
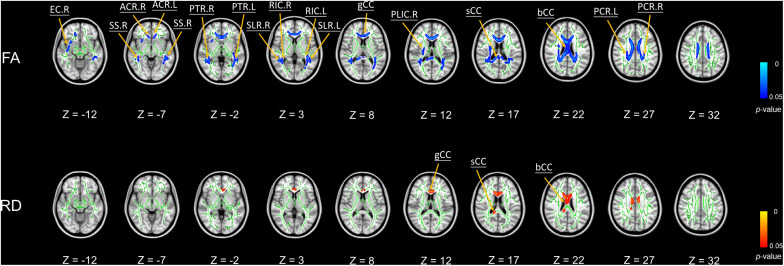
Significant voxel-wise correlations between diffusion metrics of brain white matter (WM) tracts and BDI scores using TBSS (5000 permutations, FWER at *p* < 0.05). Red color indicates the significant positive correlations between WM tracts and BDI; blue color represents the significant negative correlations. Abbreviations: FA, fractional anisotropy; RD, radial diffusivity; bCC, body of corpus callosum; sCC, splenium of corpus callosum; gCC, genu of corpus callosum; SS, sagittal stratum; PTR, posterior thalamic radiation; ACR, anterior corona radiata; RIC, retrolenticular part of internal capsule; SCR, superior corona radiata; SLF, superior longitudinal fasciculus; EC, external capsule; PLIC, posterior limb of internal capsule; R, right hemisphere; L, left hemisphere.

We did not find any significant correlations between global PSQI scores and any DTI metrics. Nevertheless, Figure 1 shows the significant negative associations of BDI scores with FA values in body, splenium and genu of the corpus callosum, bilateral sagittal stratum, bilateral posterior thalamic radiation, bilateral anterior and superior corona radiata, bilateral retrolenticular part of the internal capsule, bilateral superior longitudinal fasciculus, right external capsule, and right posterior limb of internal capsule. Furthermore, we found BDI scores were significantly positively correlated with RD values in body, splenium, and genu of the corpus callosum (Figure 1).

**FIGURE 2:**
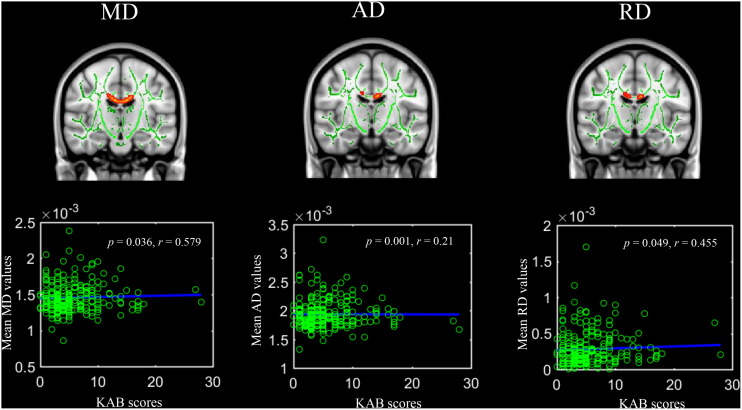
Significant voxel-wise correlations between diffusion metrics of body of corpus callosum and current life stress level (Kurzfragebogen zur Aktuellen Beanspruchung, 5000 permutations, FWER at *p* < 0.05). Red color indicates significant positive correlations.

Kurzfragebogen zur aktuellen Beanspruchung scores were significantly positively linked with MD values, AD values, and RD values in the body of the corpus callosum (Figure 2). Regarding the JHU-ICBM atlas-based correlation analysis, KAB scores were significantly positively correlated with mean MD, AD, and RD in bilateral inferior cerebellar peduncle (Right hemisphere: *r* = 0.16, *p* = 0.014; *r* = 0.18, *p* = 0.006; *r* = 0.14, *p* = 0.038; Left hemisphere: *r* = 0.19, *p* = 0.003; *r* = 0.18, *p* = 0.006; *r* = 0.13, *p* = 0.042).

**FIGURE 3:**
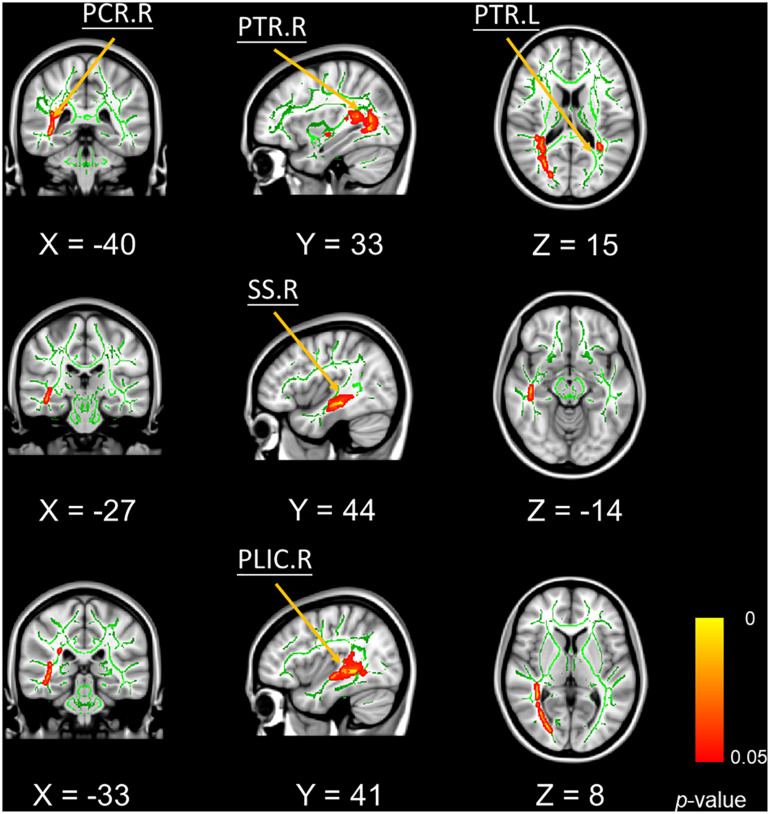
Significant voxel-wise correlations between fractional anisotropy of white matter tracts and the outcomes of visual pattern test (5000 permutations, FWER correction, *p* < 0.05). Red color indicates the significant positive correlations. Abbreviations: PTR, posterior thalamic radiation; PCR, posterior corona radiate; PLIC, petro lenticular part of internal capsule; SS, sagittal stratum; R, right hemisphere; L, left hemisphere.

In addition, mean FA values in bilateral posterior thalamic radiation, right posterior corona radiate, and right sagittal stratum were significantly positively correlated with better VPT performances (Figure 3).

### Mediation Analysis

**FIGURE 4:**
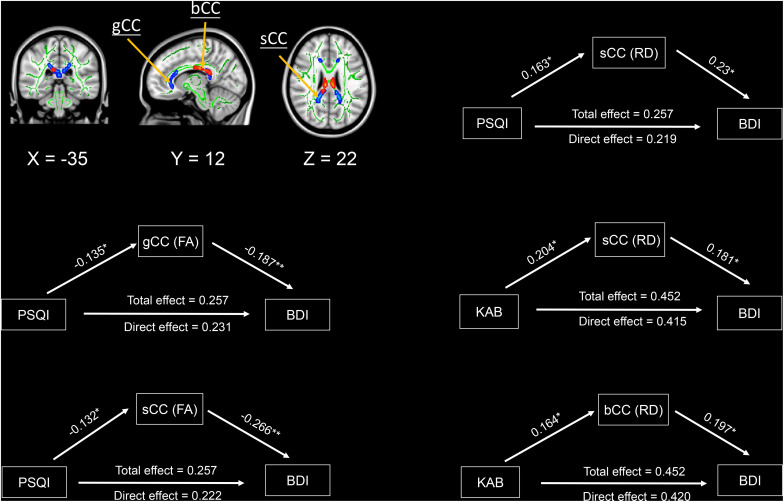
Mediation effects among sleep quality, current stress levels, brain white matter microstructures, and depressive symptomatology. Significant areas were extracted from above voxel-wise correlations analysis, which were significantly correlated with depression and stress. Green/Red colors indicate the significant negative/positive correlations between diffusion metrics and depression/stress. Significant level: * *p* < 0.05, ** *p* < 0.005. Abbreviations: FA, fractional anisotropy; RD, radial diffusivity; PSQI, Pittsburgh sleep quality index; BDI, Beck depression inventory; KAB, Kurzfragebogen zur Aktuellen Beanspruchung; sCC, splenium of corpus callosum; gCC, genu of corpus callosum; bCC, Body of corpus callosum.

The three variables mediation analysis revealed that only mean FA values in genu and splenium of the corpus callosum, as well as mean RD values in the splenium of the corpus callosum partially mediated the associations of PSQI with BDI scores at a significance level of *p* < 0.05 (Figure 4). Similarly, mean RD values in splenium and body of the corpus callosum also partially mediated the associations of KAB scores with their BDI scores (Figure 4). We found no significant mediation effects among brain WM microstructure, PSQI scores, and KAB scores.

## Discussion

In our present study, we investigated between-group differences of brain WM microstructure on sleep quality and gender differences, as well as mediation effects among sleep quality, emotional dysregulation, and WM tracts in a large sample of an aging population. First, we did not observe any significant differences in diffusion metrics between good sleepers and poor sleepers. However, the males showed significantly lower MD, AD, and RD values in the pontine crossing tract and bilateral inferior cerebellar peduncle compared to females. Further, voxel-wise correlation analysis suggested that higher depressive symptomatology was significantly negatively correlated with FA but positively related with RD in the corpus callosum, while current stress level-associated (KAB) WM tracts were positively correlated with MD, AD, and RD values in the body of the corpus callosum and the inferior cerebellar peduncle. In addition, we found that higher FA in several visual-related tracts was significantly positively correlated with the outcomes of VPT. Lastly, the mediation analysis revealed that the diffusion metrics in genu and splenium of the corpus callosum partially mediated associations of high PSQI scores/KAB scores with their high BDI scores.

In contrast to our findings, several DTI studies (Khalsa et al., 2017; Sexton et al., 2017; Amorim et al., 2018; Takeuchi et al., 2018) reported that worsened sleep quality was associated with disrupted WM tracts of frontal, temporal, occipital, and subcortical regions. It should be noted that the above studies are varying with demographics of the participants (e.g., gender and ages), sample size, MRI sequences, and the statistical methods (voxel-wise comparisons, ROI-based analysis, or structural connectivity analysis). More importantly, the components of PSQI measure (e.g., sleep duration and sleep efficiency) were affecting the reproducibility regarding the associations between sleep quality and brain WM microstructure. Although global PSQI scores have been validated for its high test–retest reliability (Knutson et al., 2006), additional objective indices of sleep quality (e.g., electroencephalography) could be helpful to address these controversies on the influence of sleep quality on brain WM microstructure. Meanwhile, we only found that pontine crossing tract and bilateral inferior cerebellar peduncle were significantly different between males and females suggesting a sexual dimorphism of the cerebellar WM microstructures, which were in line with earlier findings of several DTI and multiple shell diffusion-weighted imaging studies with a broad range of ages (Inano et al., 2011; Kumar et al., 2013; Kodiweera et al., 2016).

Our voxel-wise correlation analysis suggested that WM integrity of the corpus callosum and visual-related system (e. g. sagittal stratum and posterior thalamic radiation) is correlated with higher scores of depressive symptomatology implying their roles in emotion dysregulation and visual processing. These findings are also in line with earlier MRI research in the patients with major depressive disorder (MDD) (Kieseppa et al., 2010; Zuo et al., 2012; Chen et al., 2016; Hermesdorf et al., 2017). For instance, based on 17 DTI studies with 641 MDD patients and 581 healthy controls, Chen et al. (2016) conducted a meta-analysis and identified reduced FA in the corpus callosum extending to the left anterior limb of the internal capsule in MDD patients. Moreover, combing positive correlations between high BDI scores and increased RD values in the corpus callosum, low persistence of myelin microstructures were suggested to be a potential structural basis for high depression levels. We found that increased MD, AD, and RD in the body of the corpus callosum and inferior cerebellar peduncle were significantly correlated with current stress levels, which were also reported with the populations with high early life stress and post-traumatic stress disorders (Paul et al., 2008; O’Doherty et al., 2018). According to correlative fMRI and cytokine studies in both animals and humans (Yang et al., 2015; Benedetti et al., 2016; Sugimoto et al., 2018), these alterations might reflect elevated levels of proinflammatory cytokines, like interleukin-1α and β, tumor necrosis factor-α and interleukin-6. In summary, our correlation analyses propose that both high depression and stress levels are linked with low myelin preservation of the corpus callosum. Regarding the correlations between brain WM tracts and neuropsychological tests, we observed that FA values in visual-related WM tracts had significantly positive correlations with outcomes of the visual pattern task suggesting their roles in visual working memory performance. In short, the retrolenticular part of the internal capsule encompasses WM pathways emerging from the lateral geniculate nucleus of the metathalamus, while the posterior thalamic radiation connects the thalamus with the posterior parietal and occipital cortices.

Lastly, with mediation analysis, we identified that the diffusion metrics in the corpus callosum partially mediated the associations of sleep quality/current stress levels and depressive symptomatology. To our knowledge, prior DTI studies (Yaffe et al., 2016; Sexton et al., 2017) only investigated the effects of sleep quality on brain WM microstructures with regressing out depression or stress scores. This limitation restricted us to generalize above results for developing the therapeutic approaches in the healthcare of aging population. Using resting-state fMRI, Cheng et al. (2018) reported that increased strengths of functional connectivity, encompassing dorsolateral prefrontal cortex, precuneus, temporal cortex, anterior and poster cingulate cortex, partially mediated the associations between depressive symptomatology and sleep quality. Other DTI-related investigations (Lacerda et al., 2005; Paul et al., 2008; Won et al., 2016) identified worsened WM integrity of the corpus callosum is a critical characteristic in poor sleepers, the people with early life stress, and MDD. Given these evidences, we postulate that either the poor sleep quality or high life stress possibly exaggerates the high depression symptomatology of the older adults through an indirect effect on the structural and functional alterations of the corpus callosum. In the clinical settings, it indicates that the protective therapies for the treatment of geriatric depression is to efficiently ameliorate sleep and life stress. However, the causality flow among these variables could be confirmed by experimental designs in the patients with comorbid insomnia/high life stress and major depression disorder. In summary, our findings suggest that the WM integrity of the corpus callosum maybe act as the critical anatomical structures potentially explaining the mechanism behind the link between the poor sleep quality/high stress and higher depressive symptomatology.

Based on 1000BRAINS dataset, previous studies (Jockwitz et al., 2017a,b) suggest, with older age, both remarkably increased functional connectivity strengths in resting-state executive and frontoparietal networks, as well as declined local atrophy (measured via the local gyrification index) in the default mode network. Although we regressed out age as covariate during the statistical analysis, more investigations should be conducted to identify to what extend the age-related WM alterations influence sleep quality in older adults. Another limitation might be the time lag between global PSQI scores and acquisition of diffusion metrics of brain WM microstructure in our sample. The median time difference between questionnaire acquisition and MRI was 63 days with an interquartile range of 128 days. Nevertheless, previous research (Backhaus et al., 2002; Knutson et al., 2006) has proven high test–retest reliability and stability of the PSQI over months. Moreover, our global PSQI scores were significantly negatively correlated with the sleep-related scores (*r* = −0.697, *p* = 1.07e-30) that were derived from life quality questionnaire (Nürnberger Lebensqualitätsfragebogen), as well as significantly positively correlated with current stress levels and BDI (*r* = 0.273, *p* < 0.1.89e-05; *r* = 0.317, *p* = 5.78e-07), which were acquired on the very same day of MRI scanning. Lastly, the mean and standard deviation of global PSQI scores in our study is 8.29 and 2.99, may reflecting the insufficient sleep quality of the elderly population. Given the distribution of global PSQI scores, we therefore selected the cut-off criteria of global PSQI scores ≤6 as the good sleepers, which is slightly different with a standard score ≤5. As we did not recruit any insomnia disorders, it should be cautious to generalize our findings to other populations. To better clarify these issues, a longitudinal study would be necessary for exploring how subjective sleep quality dynamically affects the WM microstructure of human brain.

## Conclusion

Using the 1000BRAINS dataset, our results suggested that WM integrity of the pontine crossing tract and bilateral inferior cerebellar was higher in the males than females. Though no significant differences in the sleep quality were detected, the findings showed that diffusion metrics of the corpus callosum partially mediate the associations of poor sleep quality/high current stress levels with higher scores of depressive symptomatology.

## Data Availability Statement

The datasets presented in this article are not readily available because the copyright of these dataset are permitted by the German Heinz Nixdorf Recall (HNR) and 1000BRAINS project. Requests to access the datasets should be directed to s.caspers@fz-juelich.de.

## Ethics Statement

The ethical application was approved by the local Ethical Committee (University Hospital Duisburg-Essen, Germany). The patients/participants provided their written informed consent to participate in this study.

## Author Contributions

CL and JS performed the data processing and calculation. SC and SM acquired the 1000BRAINS dataset. CL and DE wrote the manuscript. NB, SL, and AB contributed to the revision of manuscript. All authors contributed to the article and approved the submitted version.

## Conflict of Interest

The authors declare that the research was conducted in the absence of any commercial or financial relationships that could be construed as a potential conflict of interest.

## Footnotes

1 https://fsl.fmrib.ox.ac.uk/fsl/fslwiki/FSL

2 http://www.neuro.uni-jena.de/cat/

3 https://www.fil.ion.ucl.ac.uk/spm/software/spm12/

4 http://stnava.github.io/ANTs/

5 https://fsl.fmrib.ox.ac.uk/fsl/fslwiki/Atlases

6 https://www.ibm.com/de-de/products/spss-statistics

7 https://www.ibm.com/us-en/marketplace/structural-equation-modeling-sem
